# Supplementary material for: On the intrinsic sterility of 3D printing
Source: PeerJ. 2016 Dec 1;4:e2661. doi: 10.7717/peerj.2661 (PMC5136128; doi:10.7717/peerj.2661)
Supplement: Table S1 — The table from Fig. 1 is provided here to make it easier to access the data within the table. [file peerj-04-2661-s002.pdf]

Method	°C	Δt
HTST	72	15s
UHT	138	2s
stovetop	63	30m
thermization	63	15s
flash	132	3m
gravity	121	30m
prevacuum	132	4m
FDM	190-240	10-120s
